# Integrating Social and Family Support as a Measure of Health Outcomes: Validity Implications from the Integrated Model of Health Literacy

**DOI:** 10.3390/ijerph20010729

**Published:** 2022-12-30

**Authors:** Anthony Faiola, Maged N. Kamel Boulos, Salman Bin Naeem, Aziz ur-Rehman

**Affiliations:** 1 Department of Health and Clinical Sciences, College of Health Sciences, University of Kentucky, Lexington, KY 40506, USA; 2 School of Medicine, University of Lisbon, 1649-028 Lisbon, Portugal; 3 Department of Library & Information Science, The Islamia University of Bahawalpur, Bahawalpur 63100, Pakistan

**Keywords:** social and family support, health literacy, healthcare, disease prevention, health promotion, health outcomes, health literacy model

## Abstract

(1) Background: Health literacy (HL) is one of the key determinants of health and healthcare outcomes. The objectives of this study are to measure and validate Sørensen et al.’s integrated model of health literacy (IMHL) in a developing country’s youth population, as well as to assess the impact of family affluence and social and family support on healthcare domains. (2) Methods: A cross-sectional survey was carried out of undergraduate university students in 19 public and private sector universities in Pakistan during June–August 2022. A nine-factor measurement model was tested using confirmatory factor analysis (CFA), and structural equation modeling (SEM) based on the 56 valid items obtained from three different validated scales, such as the family affluence scale (FAS-II), the multidimensional scale of perceived social support (MSPSS), and the European Health Literacy Questionnaire (the HLS-EU-Q). (3) Results: The data were collected from 1590 participants with a mean age of 21.16 (±2.027) years. The model fit indices indicate that the model partially fitted the data: *χ*^2^ = 4.435, df = 1448, *p* = 0.000, RMSEA = 0.048, TLI = 0.906, CFI = 0.912, IFI = 0.912, GFI = 0.872, NFI = 0.889, RFI = 0.882, PGFI = 0.791. The structural equation model showed acceptable goodness of fit indices, indicating a significant direct influence of social and family support on healthcare and disease prevention. (4) Conclusions: Social and family support are the most influential factors, with regard to HL dimensions, in improving healthcare, disease prevention, and health promotion in low-income settings and among non-English-speaking communities.

## 1. Introduction

Health literacy (HL) is a major area of interest for professionals involved in health communication, health promotion, and public health. It provides a framework for understanding the potential intersection between education and health and its role in increasing individuals’ lifelong capacities and abilities to achieve and maintain good health [1]. The term ‘health literacy’ was first coined in the 1970s [2] and was primarily seen as one’s ability to access and understand health information [3].

The concept of HL has since then evolved from educating to involving individuals, families, and communities in health and healthcare decision-making. There has never been a single, widely-accepted definition of health literacy due to the complexity of the subject. However, during the COVID-19 pandemic, a consensus on an operational definition developed among several stakeholders, including health organizations and scientists. For example, the U.S. Department of Health and Human Services defines HL as ‘the degree to which individuals have the ability to find, understand, and use information and services to inform health-related decisions and actions for themselves and others’ [4]. This definition builds on and expands the one given in Healthy People 2010 and 2020: ‘The degree to which individuals have the capacity to obtain, process, and understand basic health information and services needed to make appropriate health decisions’ [5]. The new definition emphasizes the ability to use health information instead of just processing or understanding it. Moreover, the ability to make ‘informed’ decisions at both the individual and community levels is explicitly stressed (rather than just an ability to make ‘appropriate’ decisions at the individual level). Similarly, the World Health Organization defines HL as ‘personal characteristics and social resources needed for individuals and communities to access, understand, appraise and use information and services to make decisions about health’ [6].

In the past, HL definitions have largely focused on improving the individual’s capacities to be able to seek health and disease prevention information. The importance of community health was largely ignored in these definitions, despite the fact that we live in local and global communities that continuously put us at risk for disease. We cannot attain good health if our neighbors and broader communities lack the necessary means to use the healthcare system for disease prevention or treatment. The opposite is also true: that is, we are all together better able to prevent disease when we have and make good use of universal access to healthcare. Recent examples of this include the 2003 SARS epidemic, the 2009 transmission of the novel H1N1 influenza, the 2014 Ebola virus outbreak, and the COVID-19 pandemic [5]. In terms of scope and implications, new definitions are more extensive and recognize the significance of each individual, the community, and the health of the greater public. As a result, HL includes more than just the ability to schedule one’s doctor appointment and read patient information material and prescriptions.

A health-literate society will ideally have in place mechanisms to recognize the severity of any HL problems it might face and will know how to protect individuals within the community from diseases and promote health through simple actions. However, it is also the obligation of healthcare professionals to provide understandable, simple, clear, and comprehensible health information. Everyone has a shared obligation to promote health literacy: the media, government, health professionals, health information providers, and the public. Lives depend on this [7,8].

Previous studies revealed that health literacy levels are relatively poor in Asian countries, with 75% to 82% of the population having low health literacy [9,10,11,12]. Low health literacy is now widely acknowledged to be strongly linked to less use of health resources and services, negative health outcomes, and higher healthcare costs with added burden on healthcare systems [13,14]. Pakistan, for example, continues to experience issues with low HL that frequently lead to late disease presentation, poor treatment compliance, and a lack of awareness about health and disease prevention. Pakistan is a country where the population is afflicted with several diseases of major impact, and where the healthcare system is inadequate and literacy rates are low: thus, increasing HL might have a substantial influence on people’s health outcomes [10]. Similarly, in neighboring countries such as India, at least nine out of ten adults have low HL [9]. A survey from Isfahan, Iran, showed that 79.6% of respondents have poor HL skills [11], and another study reported that one in three adults in Malaysia have low HL [12].

Several researchers employed the ‘integrated model of health literacy’ to measure the HL skills among the different population. For example, Oberne [15] in her PhD dissertation tested the IMHL among American undergraduate university students in order to predict their dietary practices. The findings showed that health literacy (HL) failed to mediate the relationship between HL determinants and dietary practices among undergraduate university students. Sukys et al. [16] measured HL skills using the European Health Literacy Survey Questionnaire (HLS-EU-Q) and reported a low health literacy level among university students in Kaunas, Klaipeda in Lithuania. The Integrated Model of Health Literacy (IMHL) has mainly been validated among chronic patients. For example, Hou et al. [17] validated IMHL in patients with breast cancer and reported that personal determinants, such as age, education, cancer duration, and stage, can significantly influence healthcare, disease prevention, and health promotion. Furthermore, Huang [18] validated the 46 items’ HLS-EU-Q, using confirmatory factor analysis (CFA) in women with breast cancer.

Attempts have been made in Asia to prepare a translated version in regional languages or a shorter version of the HLS-EU Q47. For example, Duong et al. [19] conducted a survey using the HLS-EU Q47 in six Asian Countries (Myanmar, Taiwan, Vietnam, Kazakhstan, Indonesia). As a result, a 12-item short-form HL questionnaire (HLS-SF12) was designed and validated using data from six countries. The researchers recommended the questionnaire as a useful tool for measuring the HL skills among the general population or patients in clinical settings in Asian countries. On the other hand, Dsouza, Broucke, and Pattanshetty [20] translated the HLS-EU Q16 (short version) into Hindi and the Kannada language and validated the Indian version of the questionnaire to be employed for valid and reliable measurements of HL among the Hindi and Kannada-speaking population of India. However, Duong et al. [21] reported that the data on health literacy in the population of Asian countries remains limited.

### 1.1. Theoretical Framework

The literature on HL offers several theoretical frameworks and conceptual models that attempted to contextualize, conceptualize, and theorize the concept of HL. The main purpose of these models and frameworks is to cut the guess work and make HL efforts theory based. For example, Nutbeam [22] introduced the notion of HL through actions for communication, health promotion, and education. He presented the differences between functional literacy (literacy and numeracy skills), interactive literacy (cognitive and literacy skills), and critical literacy (the application of advanced cognitive skills) in HL. He put forth a ‘health outcome model,’ emphasizing that HL is a key outcome of health education. Johnson [23] adopted the dimensions that were proposed by Nutbeam in a model of HL, in addition to dimensions such as, ‘education system’, ‘health-care system’, ‘culture/home’, and ‘community’. Paasche-Orlow and Wolf [24] proposed a model focusing on low HL and its consequences on health status and outcomes. It showed that demographic factors (age, race/ethnicity, occupation, education, income, language, culture, social support), and personal competences (vision, hearing, verbal ability, memory, and reasoning), have an impact on HL, which in turn influences healthcare access and utilization, communication between patient and healthcare provider, and self-care, all of which are ultimately involved in improving health outcomes [25]. The U.S. Institute of Medicine organized a committee of reputable academicians and researchers to examine the status of low HL in order to develop a future plan. The committee identified problems in HL, highlighted barriers to producing a health literate public, and proposed a how-to framework for examining HL among populations. The framework’s pathways show that factors such as cultural and conceptual knowledge, education, language, communication and assessment skills influence HL, which in turn affects health outcomes and costs [25].

A benchmark review study identified 12 conceptual models of HL published between 2000 and 2010 showing a wide range of variations in factors considered as key dimensions of HL [14]. For example, while some models adopted functional, interactive, and critical HL as important factors, others employed health risk behavior, disease management, self-care knowledge, cultural, language, numeracy skills, conceptual knowledge, civic literacy and media literacy, as key factors. The commonly used factors included critical skills, as well as antecedents, namely, socio-economic status, educational, psychological and environmental factors, gender, ethnicity, culture and language, income disparity, social support, and prior experience with disease. Additionally, a variety of outcomes was recorded, including the improvement of self-management abilities, enhanced access to health services, improved public health, decreased cost and burden on healthcare, and disease prevention.

**Figure 1:**
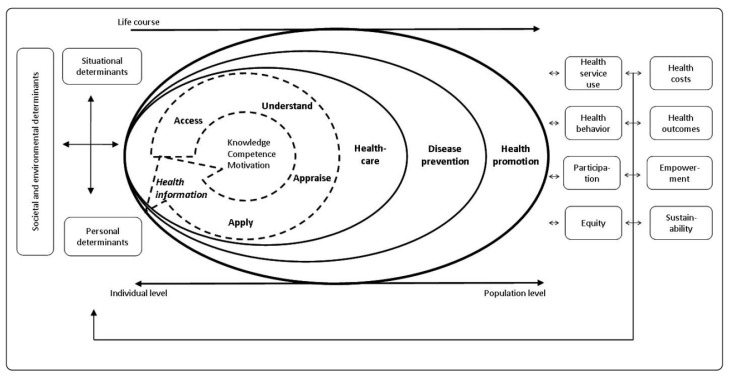
Sørensen et al.’s integrated model of health literacy (2012; courtesy of Dr. Sørensen and her colleagues under CC by 2.0 license) [14].

In their study, Sørensen et al. [14] suggested that these varied factors can be categorized into two broad dimensions, namely, the core characteristics of HL, such as ‘functional’, ‘interactive’, and ‘critical’ HL, and its scope and field of application, such as ‘patient in healthcare’. Based on their content analysis, Sørensen et al., proceeded to propose the integrated model of health literacy (IMHL) with its 12 dimensions (Figure 1). These HL dimensions refer to knowledge, motivation, and competencies in accessing, understanding, evaluating, and applying health information in the contexts of ‘healthcare’, ‘disease prevention’, and ‘health promotion’, respectively. IMHL also contains the distal and proximal factors, in addition to the components, which influence HL and indicate the pathways towards HL and health outcomes. Societal and environmental factors (demographic situation, language, societal systems, political forces, and language) are categorized under distal factors. The proximal factors focus more on personal determinants (gender, race, age, education, occupation, socio-economic status, and income) and situational factors (media use, family and peer influences, and social support).

The knowledge and skills required to navigate the three stages of the health continuum—as a patient in a medical environment (healthcare), a person at risk for disease in a prevention-of-disease system (disease prevention), and as a citizen with regard to efforts being made to promote health in the community and at workplace (health promotion)—are developed through this process. People are better able to take control of their health by using both their general and numeracy literacy skills along with their specific HL skills to access the needed information, understand it, critically evaluate it, and be able to use it and take appropriate actions, overcoming personal, economic, social and structural barriers to health by working through the stages in each of these domains of the HL process. The abilities and capacities of HL evolve throughout a person’s life and are tied to lifelong learning because contextual demands change over time and because navigating the health system depends on cognitive and psychosocial development, as well as on prior and present experiences. A shift from an individual to a population perspective is depicted by the three domain-specific frameworks (healthcare, disease prevention, health promotion). As a result, the model blends the ‘medical’ concept of HL with the wider ‘public health’ perspective, placing more attention on HL outside of the healthcare context to promote preventative health and minimize the burden on the healthcare system. Figure 1’s model also depicts the primary antecedents and consequences of HL.

**Figure 2:**
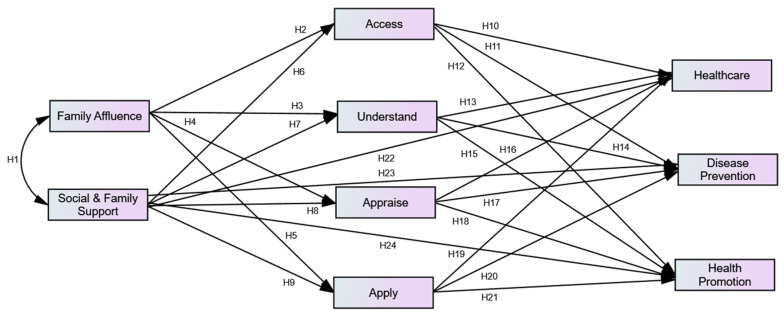
Conceptualization of hypotheses development. See Appendix A for H_1_–H_24_ descriptions.

Despite the fact that several conceptual models for HL have been published in the literature, none of them appears to have been acknowledged as being sufficiently thorough to match the changing definitions and concept of HL [14,26,27]. However, the IMHL includes components of a conceptual framework that highlights the key determinants of HL and is aligned with the new definitions of HL. The IMHL carries an experimental value in it. Moreover, only a small number of conceptual models have been validated empirically. To address these deficiencies, the IMHL, which captures the key elements of earlier conceptual models, was employed for conceptualization of the hypotheses’ development process in the present study (Figure 2).

### 1.2. Objectives of the Study

The objectives of the present study were to measure and validate the ‘integrated model of HL’ (IMHL) in the youth population in a developing country and assess the impact of family affluence and social and family support on HL dimensions. Twenty-four hypotheses were set using the different paths of the IMHL (Figure 2 and Appendix A). For example, H_1_ indicates that family affluence and social and family support are associated with each other; H_2_ shows that family affluence has an impact on accessing health information; and H_9_ indicates social and family support have an impact on applying health information.

This study is important for at least two reasons. First, a few studies in the published literature have previously measured and validated the practical value of the IMHL mainly in the population with chronic diseases. Second, there is a related lack in developing countries, particularly in Pakistan, of research testing the validation of the IMHL and investigating the different dimensions of HL and its outcomes in healthcare, disease prevention, and health promotion, as well as the impact that family affluence and social and family support are having on HL and its outcomes.

## 2. Materials and Methods

### 2.1. Participants and Procedure

A cross-sectional survey was carried out in 19 public and private sector universities in Pakistan. The study population consists of enrolled undergraduate university students (males and females), in five major disciplines: science, social sciences, arts and humanities, computer sciences, and engineering. The decision to use ‘youth’ as a study sample was made for two reasons. First, the majority of Pakistan’s population (64% of the total) is between the ages of 15 and 29 (a group we refer to as the young) [28]. Furthermore, it is important to identify youth populations who are at risk of poor health because health inequalities and health-related behaviors at this age often last into later adulthood [29].

### 2.2. Research Tools

A questionnaire was adapted based on three previously validated questionnaires, namely, family affluence scale-FAS II, multidimensional scale of perceived social support (MSPSS), and the European health literacy questionnaire (the HLS-EU-Q). These adapted questionnaires including demographic information-related questions were compiled in a four-part single questionnaire according to the purpose and need of the study (Appendix B). The first part of the questionnaire covers demographic information, such as gender, age, educational background, and type of university.

The second part consists of questions on ‘family affluence’. It contains 4 statements which are adopted from family affluence scale FAS II [30,31]. The FAS-I was developed in 1997/1998 and includes questions about owning a car, a bedroom, and taking vacations away from home to indicate the material wealth and deprivation of families. The updated FAS-II, developed in 2004, added a new question on computer ownership to FAS-I items. The health behavior of school-aged children (HBSC) project employed FAS for over a decade to examine and explain socioeconomic disparities across a range of health indicators [31]. Several studies indicated that FAS score is a predictor of health outcomes [32,33,34].

The third part is a sub-scale about ‘social and family support’ comprising 6 statements adopted from an existing 12-item ‘multidimensional scale of perceived social support (MSPSS)’ [35]. The six statements were used to measure the social and family support our participants receive from their family and friends.

The fourth part consists of a sub-scale dedicated to HL dimensions, such as accessing (6 items), understanding (7 items), appraising (7 items), and applying (7 items) health information. Three more item groups cover the domains of ‘healthcare’ (7 items), ‘disease prevention’ (5 items), and ‘health promotion’ (7 items). All these items in the fourth part were adopted from the European HL Questionnaire (the HLS-EU-Q) developed by the European HL Consortium [14]. The HLS-EU-Q was developed on the basis of the IMHL and is a widely used questionnaire to measure the HL level among various population [36,37]. The reason for using the original version of the HLS-EU-Q instead of the translated version is that the population of the study comprises university undergraduate students, and their primary language of instruction is English. Moreover, English is the official language in Pakistan for correspondence.

Overall, our questionnaire covers nine factors in 56 statements: (1) family affluence, (2) social and family support, (3) accessing information, (4) understanding, (5) appraising, and (6) applying it, in addition to the three domains of (7) healthcare, (8) disease prevention, and (9) health promotion) (Appendix B).

The reliability of the questionnaire is measured through Cronbach’s alpha. The four statements of the FAS received a 0.583 Cronbach’s alpha score, the 6 items on social and family support received a 0.868 score, and the HLS-EU-Q received the score 0.940 for 46 items. The overall 56 statements of the questionnaire received a Cronbach’s alpha value of 0.935, which indicates the high reliability of the questionnaire.

### 2.3. Data Collection and Analysis Procedure

Data for the present study were collected through purposive sampling. In total, 2500 copies of a questionnaire were distributed among the participants, of which 1590 filled questionnaires were returned with a response rate of 63.6%, and these were valid for data analysis. The good response rate was an outcome of extensive meetings involving the study’s researchers and data collection assistants regarding the adoption of useful strategies for collecting data in an effective way. As a result, a data collection team of four research assistants, all having postgraduate qualifications, visited each participating university after obtaining consent from the respective authorities to collect the data for the study. The team adopted a multi-tiered approach, such as visiting classrooms, libraries, and seminar rooms for collecting data. Team members explained the objectives and significance of the research study to participants before distributing the questionnaire to them; they also answered participants’ questions, if any. The librarians of participating institutions acted as study facilitators; their role was to lend support from their institutions in reaching out to, and collecting data from, participants.

The collected data were analyzed using the ‘statistical package for social sciences’ (SPSS software v26 by IBM) and SPSS ‘analysis of moment structures’ (AMOS). Missing values in the dataset were replaced using ‘expectation maximization’ (EM) methods. Demographic information was analyzed and presented in frequency and percentage. The chi-square statistics were applied to find the differences, if any, in the gender distribution of the respondents and their enrollment in public and private sector universities, and educational background. Confirmatory factor analysis (CFA) was employed to estimate the correlation between the latent variables and for model estimation. The structural equation model (SEM) was then employed to estimate the direct and indirect effects of different paths of the IMHL and validate the hypotheses of the study. The significance value was set at <0.05.

## 3. Results

### 3.1. Demographic Information

Demographic information revealed that of the 1590 respondents, the majority or 1046 (65.8%) were male, and 544 (34.2%) were female. The mean age of respondents was 21.16 years, with a standard deviation of 2.027, and a minimum age of 17 years and a maximum of 26 years. The majority, 1287 (80.9%), of respondents were from public sector universities, while 303 (19.1%) were from private sector universities. Respondents’ educational background was pre-medical in 728 (45.8%) participants; other educational backgrounds included a pre-engineering background (330 or 20.8%), computer science background (285 or 17.9%), background in humanities/arts (148 or 9.3%), and a background in different subjects (99 or 6.3%). Using a chi-square statistic, we found a statistically significant difference in gender of the respondents and their enrollment in public (82.7% male vs. 77.6% female) and private (16.8% male vs. 21.7% female) sector universities (*χ*^2^ = (df = 3) 9.686, *p* = 0.021). A significant difference is also found in the educational background of male and female respondents (*χ*^2^ = (df = 6) 151.580, *p* = 0.000). The majority of female respondents were from a pre-medical background (35.5% male vs. 65.6% female), as compared with males who had an educational background in pre-engineering (26.9% male vs. 9% female) and computer science (21.6% male vs. 10.8% female).

### 3.2. Confirmatory Factor Analysis

**Figure 3:**
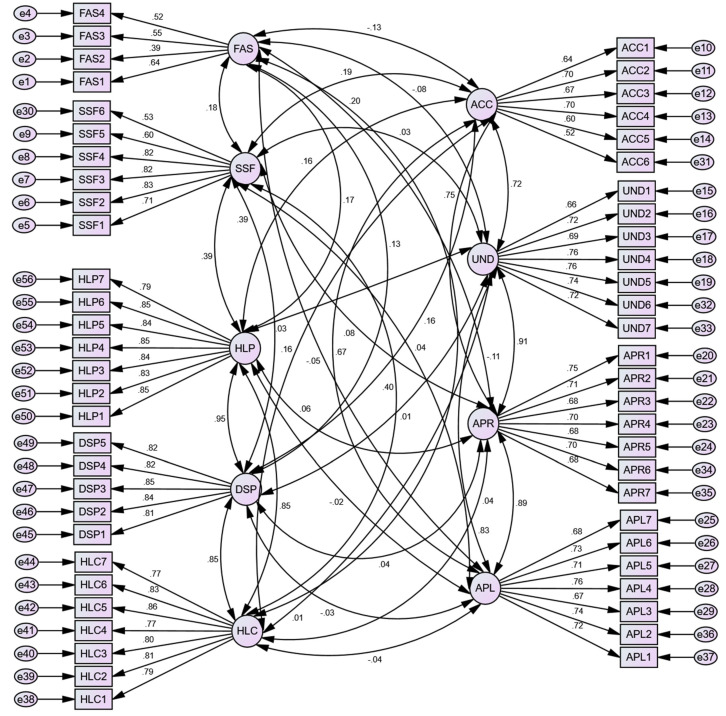
Confirmatory factor analysis (CFA) estimating the constructs. See Appendix B for detailed descriptions of family affluence status (FAS), social and family support (SSF), accessing (ACC), understanding (UND), appraising (APR), applying (APL), healthcare (HLC), disease prevention (DSP), and health promotion (HLP) items.

Figure 3 illustrates model estimation using confirmatory factor analysis. A nine-factor measurement model of family affluence status (FAS), social and family support (SSF), HL dimensions, such as accessing (ACC), understanding (UND), appraising (APR), applying (APL) health information for healthcare (HLC), disease prevention (DSP), and health promotion (HLP) was tested using CFA, based on the 56 valid items obtained from the three different scales in our questionnaire (Appendix B).

Model fit indices were used to evaluate the initial estimating model, e.g., chi-square, degree of freedom (df), root mean square error of approximation (RMSEA), the Tucker–Lewis index (TLI), comparative fit index (CFI), incremental fit index (IFI), and modification indices, loadings, covariances, and correlations. The model fit indices showed the model only partially fits the data: *χ*^2^ = 4.435, df = 1448, *p* = 0.000; RMSEA = 0.048; TLI = 0.906, CFI = 0.912, IFI = 0.912, GFI = 0.872, NFI = 0.889, RFI = 0.882, PGFI = 0.791. The chi-square value of 4.435 p = 0.000 is higher than the accepted value (≤2 or 3) and shows a significant difference between the observed and proposed model. Moreover, the GFI value = 0.872, which is considered as one of the most important indices in the goodness of fit model, is lower than the accepted value of >0.9.

#### 3.2.1. Standardized Estimation of Regression Weights

The standardized estimation of regression weights of the factors and their loadings in the confirmatory factor analysis are shown in Figure 3. The path coefficients values of the latent variables (FAS, SSF, ACC, UND, APR, APL, FAS, SSF, HLC, DSP, HLP) are moderate to high, ranging from *β = *0.39 to *β = *0.85. The latent variables ‘family affluence status’ (FAS) and ‘social and family support’ (SSF) are measured using 4 and 6 observable variables, respectively. The values of the 6 items on SSF range from *β = *0.53 to *β = *0.71, which shows a strong association of the loadings. The loadings on the FAS range from *β = *0.39 to *β = *0.64, also suggesting a strong association, with the exception of the FAS2 item, which has *β = *0.39, a medium association. The loadings on the four dimensions of HL, namely, access (ACC), which has six loadings (value ranges between *β = *0.599 to *β = *0.700); understanding (UND) with seven loadings (ranging between 0.66 to 0.76); appraisal (APR), also with seven loadings (value ranges between *β = *0.68 to *β = *0.74), and apply (APL), again with seven loadings (values ranges between 0.67 to 0.75), demonstrate a strong relationship between the loadings on the relevant latent variables. Three loadings on the other three latent variables, namely, healthcare (HLC), disease prevention (DSP), and health promotion (HLP), range between *β = *0.78 to *β = *0.85, indicating strong association of the loadings (Figure 3).

#### 3.2.2. Standardized Estimation of Correlation among the Latent Variables

Figure 3 presents the correlation estimation among the nine latent variables that formed the basis of the confirmatory factor analysis. The correlation value (*β = *0.176) indicates a small strength positive correlation between family affluence status (FAS) and social and family support (SSF). However, FAS is negatively correlated with ACC (*β = *−0.1290), UND (*β = *−0.083), APR (*β = *−0.110), and APL (*β = *−0.045) (accessing, understanding, appraising, and applying healthcare information). The strongest correlation is found between DSP and HLP (*β = *0.953), HLC and DSP (*β *=**0.854), and HLP (*β = *0.848). SSF is correlated with HLC (*β = *0.396), DSP (*β = *0.385), and HLP (*β = *0.395), more than with the latent variables related to HL dimensions, such as ACC, UND, APR, and APL. A negative correlation exists between APL information for HLC (*β = *−0.036), DSP (*β = *−0.026), and HLP (*β = *−0.20) (applying information for healthcare, disease prevention, and health promotion).

### 3.3. Structural Equation Model

**Figure 4:**
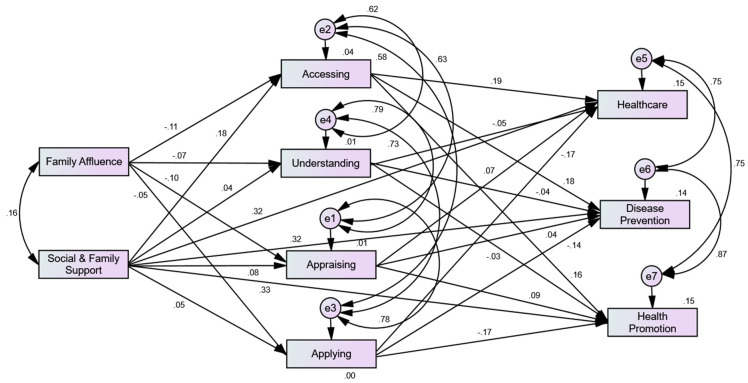
Structural equation model estimating the direct and indirect effects.

Figure 4 depicts the structural equation model estimating the direct and indirect effects. A nine-factor structural equation model was used to estimate the ‘integrated model of health literacy (IMHL)’. In order to present the computed factors, 56 observed variables (Figure 3) were summed up using SPSS. The correlation value indicates that family affluence is positively associated with social and family support (*β = *0.16). Family affluence has a negative influence on accessing (*β = *−0.11), understanding (*β = *−0.07), appraising (*β = *−0.10), and applying (*β = *−0.05) healthcare information. According to path coefficient estimation, there is a strong association between the dimensions of HL. For example, accessing healthcare information is strongly associated with understanding (*β = *0.62), appraising (*β = *0.63), and applying (*β = *0.58) healthcare information. Similarly, the values indicate that healthcare is strongly associated with disease prevention (*β = *0.75) and health promotion (*β = *0.75). Disease prevention is also strongly correlated with health promotion (*β = *0.87).

#### 3.3.1. Squared Multiple Correlations

The squared multiple correlation values showed that factors such as family affluence, social and family support, and HL dimensions together account for 15% of the variance in individual healthcare (*R* = 0.15), 14% of the variance in disease prevention (*R* = 0.14), and 15% of the variance in health promotion (*R* = 0.15). On the other hand, only a 4% variance in accessing, 1% in understanding, and 1% in appraising information are accounted for by the combined effect of family affluence and social and family support (Figure 4).

#### 3.3.2. Standardized Indirect, Direct, and Total Effects

The structural equation model was also estimated with three additional paths, including social and family support to healthcare, disease prevention, and health promotion (Figure 4). The estimation indicates that family affluence has negative indirect effects, if dimensions of HL, such as accessing, understanding, appraising, and applying, mediate the relationship between family affluence and healthcare (*β = *−0.015), health promotion (*β = *−0.015), and disease prevention (*β = *−0.013). On the other hand, the positive indirect effects of social and family support on healthcare (*β =* 0.027), health promotion (*β =* 0.025), and disease prevention (*β = *0.025), are quite low, as compared with direct effects on healthcare (*β =* 0.32), health promotion (*β =* 0.33), and disease prevention (*β =* 0.32). In sum, the standardized total effects of family affluence are negative on healthcare (*β =* −0.015), health promotion (*β =* −0.015), and disease prevention (*β =* −0.013). On the other hand, we note the total positive standardized effects (medium size strength) of social and family support on individual healthcare (*β =* 0.35), health promotion (*β =* 0.357), and disease prevention (*β =* 0.347) (Table 1). However, these effects are highest on healthcare, disease promotion, and disease prevention, as compared with any other factor used in the model. This shows that individual and community healthcare, disease prevention, and health promotion are more influenced by family and social support, as compared with HL skills (Table 1 and Figure 4).

#### 3.3.3. Model Fit Indices

The measures such as absolute fit (*χ*^2^, GFI, RMSEA) and incremental fit (TLI, NFI, RFI, IFI, CFI, RFI) are used to test the model fit. The recommended values are CMIN/df ≤ 3, GFI ≥ 0.90, CFI ≥ 0.90, NFI ≥ 0.90, RFI ≥ 0.90, TLI ≥ 0.90, RMSEA ≤ 0.08 [38].

The model fit indices showed acceptable goodness of fit values of: *χ*^2^ (df = 3) = 30.299; *p* = 0.000; GFI = 0.996, IFI = 0.997, TLI = 0.962, CFI = 0.997, NFI = 0.997, RFI = 0.958, PGFI = 0.066, RMSEA = 0.076.

#### 3.3.4. Standardized Estimation of Regression Weights and Validation of the Hypotheses

Table 2 presents the standardized regression estimates, standard error (SE) for the estimation of parameter, critical ratio (CR), significance among factors, and validation of the hypotheses. Regression is a measure that allows researchers to predict the variation in one variable based on another variable. The regression results of the latent variables showed a significant relationship. The significance level is set at *p* = 0.05, and the symbol ‘***’ denotes the likelihood that the variable’s value will not exceed the critical threshold of 0.005. This implies that the sample data establish the curve’s rules. The findings indicate that family affluence is significantly associated with social and family support (*β* = 1.949, CR = 6.134, *p* < 0.000). However, family affluence is negatively associated with accessing (*β* = −0.0251, CR = −4.490, *p* < 0.000), understanding (*β* = −0.212, CR = −2.902, *p* < 0.05), appraising (*β* = −0.267, CR = −0.3879, *p* < 0.000), and applying (*β* = −0.150, CR = −2.109, *p* < 0.05) healthcare information (Table 2).

Social and family support is significantly positively associated with accessing (*β* = 0.134, CR = 7.059, *p* < 0.000), appraising (*β* = 0.074, CR = 3.153, *p* < 0.05), and applying (*β* = 0.048, CR = 1.981, *p* < 0.05) health information. It is also associated with the individual’s healthcare (*β* = 0.388, CR = 13.71, *p* < 0.000), disease prevention (*β* = 0.259, CR = 13.58, *p* < 0.000), and health promotion (*β* = 0.364, CR = 14.10, *p* < 0.000). However, social and family support is not associated with the individual’s understanding (*β* = 0.043, CR = 1.740, *p* > 0.05) of health information. There is a statistically significant influence of health information accessing on healthcare (*β* = 0.292, CR = 5.857, *p* < 0.000), disease prevention (*β* = 0.186, CR = 5.534, *p* < 0.000), and health promotion (*β* = 0.224, CR = 4.931, *p* < 0.000). However, information understanding, appraising, and applying have either statistically no influence or negative influence on healthcare, disease prevention, and health promotion. However, only appraising information has a positive influence on health promotion (*β* = 0.109, CR = 2.091, *p* < 0.05)

## 4. Discussion

Developing adequate health literacy skills, particularly among young people (which form a large majority of the population in developing countries), can effectively address health disparities, improve public health, and foster both individual and community resilience. Such skills can also encourage and empower individuals to take a more active role in health promotion. People with good HL skills are typically better equipped to control their health than those with inadequate skills [7]. Inequalities in access to, and delivery of, healthcare services, poor levels of HL competencies, and uneven distribution of HL training programs across rural and urban areas, developed, developing, and underdeveloped countries, English and non-English speaking communities, all pose a threat to global health.

Our study supports and confirms the findings of other studies that family affluence does not predict the level of HL [39,40]. However, a number of studies revealed that among school-aged children, family affluence is linked to a greater degree of HL [41,42,43]. Our findings revealed a very useful insight that social and family support directly influence access to healthcare, disease prevention, and health promotion. These findings support the results of other studies that identified social support as a significant determinant of self-care behaviors [44,45,46,47,48]. The IMHL (Figure 1) illustrates the influence of social support on health domains through the paths of HL dimensions. Our findings show that the direct influence of social support on health domains is more significant than the indirect effect. These results are encouraging in that they point the way toward creating an updated framework for individuals and communities with lower levels of general literacy and HL to empower them through family and social support, which in turn should promote their healthy behaviors, improve their use of healthcare and self-care, and enable them to better protect themselves from disease (disease prevention and health promotion). The concept of health promotion has evolved over time, moving from one that placed greater emphasis on the need for medical professionals to educate the public to one that emphasizes community involvement in decision-making, program design, and evaluation [49,50]. The results of our study therefore point to possible strategies that might be in line with this evolution of the concept of health promotion, putting more focus on social support and family involvement in healthcare.

Of the 24 hypotheses (Appendix A), 13 hypotheses were rejected in the study (Table 2). This demonstrates that more than half of the path coefficients in the proposed model were not supported by the observed model. One of our findings showed that applying health information has a negative non-significant impact on individual health domains, such as healthcare, disease prevention, and health promotion. In contrast, many research studies reported a significant impact of applying health information on health outcomes [7,51,52].

Our findings also revealed a non-significant negative impact of understanding and appraising health information on healthcare and disease prevention. These results can be correlated with applying health information. One aspect behind this negative effect could be that respondents’ actual competency and ability to ‘understand’ and ‘appraise’ information is not always the same as their perceived competency and ability. In fact, understanding and appraising health information is not straight forward; it requires a range of skills, including the ability to critically examine the authenticity of the information, its accuracy, relevance, and appropriateness, as well as the legitimacy and trustworthiness of the source of health information. The application of incorrect, outdated, or inappropriate information often results in a negative health outcome. Therefore, different strategies are required to improve HL among populations in developing countries. Studies have shown that educational attainment (general literacy) is strongly correlated with HL levels [53,54], but the concept of HL seems less complex in English-speaking countries where the majority of health information, medical instructions, drug leaflets, etc., are written in English, as opposed to countries where English is not spoken as the first language, where there is paucity of suitable health information in local languages [55], and where general literacy is lower [56,57].

In developed countries, adult general literacy rates are typically 96% or higher, compared with an average literacy rate of 65% in developing nations [58]. HL statistics also reflect the general literacy ratio. For instance, 52% of adults in the US, 47% in Europe, 43% in the UK, 60% in Canada, and 60% in Australia, had low HL [1,59,60] compared with 82.4%, 90%, and 79.6% of adults in Pakistan, India, and Iran, respectively [9,11,61]. As a result, the populations of developing countries are more exposed to, susceptible to, and burdened by both communicable and non-communicable diseases [62,63]. Poor literacy levels can be improved by providing access to better school education. By attaining good literacy levels, literate societies will be better prepared to make significant contributions to community health [64].

### 4.1. Implications of the Study

The study has some important theoretical and practical implications. It measured and validated the integrated model of health literacy (IMHL) in a population from a developing country, Pakistan. The findings validate the model paths and variables within a construct. However, the data do not validate the connection from one construct to another; for example, applying information has no influence on healthcare domains in the model. The validation findings showed direct paths from social and family support to healthcare domains. These three newly proposed paths are the most significant predictors in the whole model; therefore, a revised model is presented as shown in Figure 4. Moreover, the population of the study has different characteristics in terms of demographics, environment, and locations compared with the populations of other studies in the domain of HL [17,65,66].

To sum up, the authors suggest the inclusion of social and family support interventions to empower individuals and communities to have increased access to, and use of, healthcare services. As a result, they will be able to navigate adverse disease events and better engage in health promotion activities.

### 4.2. Limitations of the Study

This study carries some limitations. The data collection tool relies on self-reported responses and could therefore be subject to respondents’ perceived ability to properly understand question statements (and the rating of this perceived ability while recording responses). Personal bias can creep in. Although the researchers used a validated questionnaire containing close ended-questions, such questionnaires have always had some degree of limitation. Moreover, it was noted that most respondents were previously unaware of the concept of HL, and had never been involved in applying health information when making healthcare decisions, even though they were informed about the objectives of the study and the concept of the HL. Hence, there is the possibility that these issues may have had an impact on the findings.

Finally, it is important to note that the findings of this study can be safely generalized across the studied country among undergraduate and graduate students. However, care should be exercised while generalizing the findings to different age and educational attainment groups, such as older people and people with lower educational achievements.

## 5. Conclusions

In the past, health literacy interventions were largely focused on improving the individual’s abilities to seek, appraise, understand, and apply health information effectively in order to gain and promote good health. The importance of community health was largely overlooked. This study reported a non-significant correlation between health literacy dimensions and healthcare domains in the surveyed population (university students in Pakistan). The authors went on to propose a revised model that highlights the significant direct influence of social and family support on healthcare and disease prevention, as documented in the study population. In conclusion, social and family support were found to be the most influencing factors, with regard to health literacy dimensions, in improving healthcare, disease prevention, and health promotion in low-income settings, and among non-English speaking communities. The study suggests conducting a quantitative meta-analysis to compare with benchmarks of work previously done in order to determine the correlation among social and family support and healthcare outcomes, such as disease prevention and health promotion. The evidence from the meta-analysis will further suggest the strength of the correlation and the descriptions of its importance to healthcare. The study further suggests identifying new models such as the use of mobile apps and digital health models to enhance the family and community support and participation in the healthcare decision-making process that can result in a healthy society.

## Figures and Tables

**Table 1:** Standardized Total Effects.

	Social & Family Support	Family Affluence	Apply	Appraise	Understanding	Access
Apply	0.050	−0.053				
Appraise	0.080	−0.098				
Understanding	0.044	−0.073				
Access	0.176	−0.112				
Healthcare	0.350	−0.015	−0.174	0.072	−0.048	0.185
Health promotion	0.357	−0.015	−0.173	0.092	−0.027	0.156
Disease prevention	0.347	−0.013	−0.144	0.043	−0.038	0.176

**Table 2:** Validation of the hypotheses.

Hypotheses	Factor		Factor	Estimate	S.E.	C.R.	* p *	Results
H_1_	Family Affluence	<-->	Social & Family Support	1.949	0.318	6.134	***	Accepted
H_2_	Family Affluence	-->	Access	−0.251	0.056	−4.490	***	Rejected
H_3_	Family Affluence	-->	Understand	−0.212	0.073	−2.902	0.004	Rejected
H_4_	Family Affluence	-->	Appraise	−0.267	0.069	−3.879	***	Rejected
H_5_	Family Affluence	-->	Apply	−0.150	0.071	−2.109	0.035	Rejected
H_6_	Social & Family Support	-->	Access	0.134	0.019	7.059	***	Accepted
H_7_	Social & Family Support	-->	Understand	0.043	0.025	1.740	0.082	Rejected
H_8_	Social & Family Support	-->	Appraise	0.074	0.023	3.153	0.002	Accepted
H_9_	Social & Family Support	-->	Apply	0.048	0.024	1.981	0.048	Accepted
H_10_	Access	-->	Healthcare	0.292	0.050	5.857	***	Accepted
H_11_	Access	-->	Disease Prevention	0.186	0.034	5.534	***	Accepted
H_12_	Access	-->	Health Promotion	0.224	0.045	4.931	***	Accepted
H_13_	Understand	-->	Healthcare	−0.058	0.050	−1.172	0.241	Rejected
H_14_	Understand	-->	Disease Prevention	−0.031	0.033	−0.926	0.355	Rejected
H_15_	Understand	-->	Health Promotion	−0.030	0.045	−0.658	0.510	Rejected
H_16_	Appraise	-->	Healthcare	0.093	0.057	1.631	0.103	Rejected
H_17_	Appraise	-->	Disease Prevention	0.038	0.039	0.979	0.328	Rejected
H_18_	Appraise	-->	Health Promotion	0.109	0.052	2.091	0.036	Accepted
H_19_	Apply	-->	Healthcare	−0.220	0.049	−4.495	***	Rejected
H_20_	Apply	-->	Disease Prevention	−0.122	0.033	−3.702	***	Rejected
H_21_	Apply	-->	Health Promotion	−0.199	0.045	−4.456	***	Rejected
H_22_	Social & Family Support	-->	Healthcare	0.388	0.028	13.71	***	Accepted
H_23_	Social & Family Support	-->	Disease Prevention	0.259	0.019	13.58	***	Accepted
H_24_	Social & Family Support	-->	Health Promotion	0.364	0.026	14.10	***	Accepted

The significance level is set at *p* = 0.05, and the symbol ‘***’ denotes the likelihood that the variable’s value will not exceed the critical threshold of 0.005.

## Data Availability

The core data supporting the findings of this study are available within the article and its appendices; further details can be obtained from the authors upon reasonable request.

## Appendix A. Hypotheses of the Study

**H_1_.** *Family affluence and social and family support are associated with each other*.

**H_2_.** *Family affluence has an impact on accessing health information*.

**H_3_.** *Family affluence has an impact on understanding health information*.

**H_4_.** *Family affluence has an impact on appraising health information*.

**H_5_.** *Family affluence has an impact on applying health information*.

**H_6_.** *Social and family support has an impact on accessing health information*.

**H_7_.** *Social and family support has an impact on understanding health information*.

**H_8_.** *Social and family support has an impact on appraising health information*.

**H_9_.** 
*Social and family support has an impact on applying health information.*

**H_10_.** *Knowledge, competence, and motivation in accessing health information has an impact on healthcare*.

**H_11_.** *Knowledge, competence, and motivation in accessing health information has an impact on disease prevention*.

**H_12_.** *Knowledge, competence, and motivation in accessing health information has an impact on health promotion*.

**H_13_.** *Knowledge, competence, and motivation in understanding health information has an impact on healthcare*.

**H_14_.** *Knowledge, competence, and motivation in understanding health information has an impact on disease prevention*.

**H_15_.** *Knowledge, competence, and motivation in understanding health information has an impact on health promotion*.

**H_16_.** *Knowledge, competence, and motivation in appraising health information has an impact on healthcare*.

**H_17_.** *Knowledge, competence, and motivation in appraising health information has an impact on disease prevention*.

**H_18_.** *Knowledge, competence, and motivation in appraising health information has an impact on health promotion*.

**H_19_.** *Knowledge, competence, and motivation in applying health information has an impact on healthcare*.

**H_20_.** *Knowledge, competence, and motivation in applying health information has an impact on disease prevention*.

**H_21_.** *Knowledge, competence, and motivation in applying health information has an impact on health promotion*.

**H_22_.** *Social and family support has an impact on healthcare*.

**H_23_.** *Social and family support has an impact on disease prevention*.

**H_24_.** 
*Social and family support has an impact on health promotion.*

## Appendix B. Items under Nine Constructs of Integrated Model of Health Literacy

**Construct**	**Items**	**Measure**	**Sources**
**Family Affluence Scale (FAS II)**			
	FAS1	Does your family own a car or another motorized vehicle?	[30,31]
	FAS2	Do you have your own bedroom?	
	FAS3	How many computers (including laptops and tablets) does your family own?	
	FAS4	How many times did your and your family travel out of the city for holiday/vacation last year?	
**Social support from family and friends**			
	SSF1	I can talk about my problems with my family	[35]
	SSF2	My family really tries to help me	
	SSF3	I get emotional help and support, when i need from my family	
	SSF4	My family is willing to help me make decisions	
	SSF5	My friends really tries to help me	
	SSF6	I can count on my friends when things go wrong	
**Access**			
	ACC1	To access information about symptoms of illness that concerns you	[14,37]
	ACC2	To access information on treatment of illness that concerns you	
	ACC3	To access information what to do in case of emergency	
	ACC4	To access where to get professional help when you are ill (such as doctor, pharmacist, psychologist)	
	ACC5	To access information about how to manage unhealthy behavior such as smoking, or low physical activity	
	ACC6	To find out about political changes that may affect health (instructions: legislation, new healthy screening programmes, changing of government restructuring of health services, etc.)	
**Understand**			
	UND1	To understand what your doctor says to you	
	UND2	To understand the leaflets that comes with your medicines	
	UND3	To understand what to do in medical emergency	
	UND4	To understand your doctor or pharmacist instruction on how to take a prescribed medicine	
	UND5	To understand health warnings about behavior such as smoking, low physical activity and drinking too much	
	UND6	To understand information on food packaging	
	UND7	To understand information in the media on how to get healthier? (instructions: internet, newspaper, magazine)	
**Appraise**			
	APR1	To evaluate how information from your doctor applies to you	
	APR2	To evaluate the advantages and disadvantages of different treatment options	
	APR3	To evaluate when you may need to get a second opinion from another doctor	
	APR4	To evaluate when you need to go to a doctor for a check-up	
	APR5	To evaluate if the information on health risks in the media is reliable (instruction: TV, Internet, or other media)	
	APR6	To appraise where your life affects your health and well-being? (instructions: your community, neighborhood)	
	APR7	To appraise how your housing conditions help you to stay healthy	
**Apply**			
	APL1	To follow the instructions that doctor gives you.	
	APL2	To call an ambulance in an emergency	
	APL3	To decide how you can protect yourself from illness based on advice from family and friends	
	APL4	To decide how you can protect yourself from illness based on information in the media (instructions: newspapers, leaflets, Internet or other media?)	
	APL5	To make decisions to improve your health	
	APL6	To influence your living conditions that affects your health and wellbeing (instructions: drinking and eating habits, exercise, etc.)	
	APL7	To take part in activities that improves health and well-being in your community	
**Health Care**			
	HLC1	To pay attention to your health	
	HLC2	To eat regularly (breakfast, lunch and dinner)	
	HLC3	To exercise regularly	
	HLC4	To get regular medical care for prevention	
	HLC5	To get medical care when needed	
	HLC6	To take time off when sick	
	HLC7	To get enough sleep	
**Disease Prevention**			
	DSP1	To prevent and better manage ill-health	
	DSP2	Changed social action for health	
	DSP3	To influence other towards health decisions	
	DSP4	Changing personal behaviors	
	DSP5	Increased used of preventive health services	
**Health Promotion**			
	HLP1	Better health outcomes	
	HLP2	Ability to manage long term conditions	
	HLP3	Creating supportive environment	
	HLP4	Strengthen community actions	
	HLP5	Developing personal skills and reorienting the health system	
	HLP6	Influence others towards health decisions	
	HLP7	Allow healthcare organizations to determine what, exactly, they aim to accomplish and how they can provide the standard of care their patients deserve	
